# Circadian clocks and memory: time-place learning

**DOI:** 10.3389/fnmol.2013.00008

**Published:** 2013-04-11

**Authors:** C. K. Mulder, M. P. Gerkema, E. A. Van der Zee

**Affiliations:** ^1^Department of Molecular Neurobiology, University of GroningenGroningen, Netherlands; ^2^Department of Chronobiology, University of GroningenGroningen, Netherlands

**Keywords:** learning, circadian, memory, aging, time, place, Cry, clock genes

## Abstract

Time-Place learning (TPL) refers to the ability of animals to remember important events that vary in both time and place. This ability is thought to be functional to optimize resource localization and predator avoidance in a circadian changing environment. Various studies have indicated that animals use their circadian system for TPL. However, not much is known about this specific role of the circadian system in cognition. This review aims to put TPL in a broader context and to provide an overview of historical background, functional aspects, and future perspectives of TPL. Recent advances have increased our knowledge on establishing TPL in a laboratory setting, leading to the development of a behavioral paradigm demonstrating the circadian nature of TPL in mice. This has enabled the investigation of circadian clock components on a functional behavioral level. Circadian TPL (cTPL) was found to be *Cry* clock gene dependent, confirming the essential role of *Cry* genes in circadian rhythms. In contrast, preliminary results have shown that cTPL is independent of *Per* genes. Circadian system decline with aging predicts that cTPL is age sensitive, potentially qualifying TPL as a functional model for episodic memory and aging. The underlying neurobiological mechanism of TPL awaits further examination. Here we discuss some putative mechanisms.

## Introduction

Time–place learning (TPL) refers to the ability to secure resources when they are available under specific temporal and spatial contingencies (Crystal, 2009). Many environmental aspects show circadian variation. Predators often establish hunting routes and many resources, like food and mates only become available on certain times of the day (Daan and Koene, 1981; Rijnsdorp et al., 1981; Silver and Bittman, 1984). This given, TPL is believed to be functional in optimizing resource localization and exploitation as well as predator avoidance in a circadian (predictable) changing environment, decreasing energy expenditure, and increasing survival chances. Although this is mainly a hypothetical explanation as to why animals possess TPL ability, an evolutionary relevance is strengthened by the fact that TPL has been shown in many species including bees (Gould, 1987), ants (Harrison and Breed, 1987), fish (Reebs, 1996), birds (Krebs and Biebach, 1989), rats (Boulos and Logothetis, 1990), and recently mice (Van der Zee et al., 2008).

The reason why animals are capable of TPL is, however, not the focus of this review, but rather what we can learn from this behavior in neuroscience. Evidence suggests that TPL can depend on the circadian system. This circadian TPL (cTPL) suggests a link between the circadian system and associative memory. However, not much is known about this connection.

The notion that time of day can be relevant in cognitive functions arose long before the concept of circadian clock systems was established. Beling published her first study on “Zeitgedachtnis,” time memory, studying sun compass orientation in honey bees (Beling, 1929). In 1950, Kramer did rather similar experiments in starlings. He showed that these birds also use an internal time of day mechanism to select the appropriate orientation relative to the position of an artificial sun (Kramer, 1950). These findings, of a functional internal timing in vertebrates, helped in the breakthrough of biological clock concepts (Aschoff, 1954; Pittendrigh et al., 1958). Since then we gained much insight into the physiology and molecular determinants of the circadian system itself and on the manifold behavioral and physiological processes under circadian control.

In mammals, a central nervous system based pacemaker, located in the suprachiasmatic nuclei (SCN), tracks light, and dark information and is connected with many peripheral, organ based oscillators (Dibner et al., 2010). In external 24 h light-dark (LD) cycles the circadian system results in entrained daily rhythms in the body. In constant external conditions, these rhythms show a free running pattern of about 24 h (circadian). The circadian sleep-wake cycle, regulation of hormonal, body temperature, and feeding rhythms are well-known examples. General cognitive- and memory performances have also been shown to vary over the circadian cycle (for review see Carrier and Monk, 2000). In line with this, disruption of circadian rhythms due to age, shift work, and shifts of the LD cycle (jet lag) have been associated with impairments of cognitive function (Fekete et al., 1985; Folkard et al., 1985a,b; Antoniadis et al., 2000; Devan et al., 2001; Biemans et al., 2003; Cain et al., 2004b; Craig and McDonald, 2008).

Besides governing innate rhythms in physiology and behavior, it is believed that circadian oscillators can provide phase information to brain systems involved in cognition, like memory, which allows time to be used in adaptive mechanisms (Enright, 1970, 1975; Gallistel, 1990; Mistlberger et al., 1996). It is believed that time of day information derived from an internal oscillator can be “stamped” in memory as a contextual feature to form associations with other contextual features and to be used in decision making processes. Such a mechanism can only function when a clock can be consulted continuously to record time-stamps, and to check whether coded time-stamps match the actual time of the day. This “continues consulted clock” function is thought to underlie cTPL as well as the adaptive time of day compensation in sun compass navigation demonstrated in several insect and bird species (Frisch, 1950; Kramer, 1950; Hoffmann, 1960; Keeton, 1974; Budzynski et al., 2000; Merlin et al., 2009).

### Terminology

In order to avoid confusion, it is necessary to explain/define some terminology regarding TPL. TPL is also referred to in the literature as time-place discrimination or time-place association. Two types of TPL paradigms can be found in the literature: Interval- and daily TPL paradigms. *Interval TPL paradigms* are specifically designed to study interval timing (the ability to keep track of elapsed time). In such paradigms, animals typically receive a single test session each day (with multiple trials/location switches), and these sessions can occur at varying times of the day. For example, animals can learn to anticipate the switch when first food is provided only at location “A,” while after “X” amount of time food is provided only at location “B.” Rats can learn an interval time-place task with at least four different feeding locations and with unequal intervals between rewarding locations (Thorpe and Wilkie, 2002). In *daily TPL paradigms*, typically the location of a resource depends on the time of day, and animals are trained over multiple days with multiple sessions per day on fixed time-points, so that they learn to visit or avoid specific locations on specific times of the day.

In this review we focus on daily TPL. Thus, when we refer to TPL, more specifically we refer to daily TPL. In the literature, daily TPL is sometimes referred to as cTPL, however, here when we refer to cTPL we refer to daily TPL with the use of a circadian strategy. Animals may use multiple strategies to solve a daily TPL paradigm, as will be explained in the next paragraph.

### Multiple possible strategies for TPL

We have mentioned that TPL can depend on the circadian system (in this case we refer to cTPL). This is only true if animals use a so called *circadian strategy* to master the paradigm. Alternatively, three non-cTPL strategies have been identified (Carr and Wilkie, 1997). These strategies will be explained here along with methods to identify these non-circadian strategies.

First, animals may use the so called *contextual cue strategy*. This is a non-timing strategy by which animals simply learn to visit or avoid location A in the presence of one (set of-) contextual cue(s), while they learn to visit/avoid location B in the presence of another (set of-) contextual cue(s). To exclude the possibility for animals to use this strategy, any discriminating contextual cues (differences between sessions) should be excluded by a proper research setup and practice.

Second, animals may use an *ordinal strategy*. In this case animals remember the sequence of (daily) events, e.g., first visit location A, then B. This strategy is also referred to as an alternation strategy and can also be viewed as the establishment of a (daily) route. Two variants of the ordinal strategy are identified: A timing- and a non-timing variant. In case of the timing variant, the sequence is reset daily, while this is not the case in the non-timing variant. The use of an ordinal strategy can be identified by skipping sessions. Note however that skipping the last session of a day will not identify an ordinal strategy when the timing variant is utilized. Animals will show normal location visits in the first session of the next day because the sequence has been reset. Thus, to identify the use of an ordinal strategy, without further distinction between the two variants, the first session(s) of a day need to be skipped. Instead of visiting the second session location, animals will visit the wrong (skipped) first session location in case an ordinal strategy is used.

Third, animals may use an *interval timing strategy*. In this case specific delay periods relative to one or more external cues are encoded as discriminating cues to know which locations to visit or avoid. For instance, animals may learn that short (or concrete after X amount of time) after lights-on, they should visit location A, while longer after lights on (or concrete after Y amount of time) they should visit location B. Because external cues can start/stop/reset timing, interval timing is also referred to as a stopwatch like mechanism. Interval timing is thought to be utilized to track intervals in a second-to-minutes range (Wilkie, 1995; Buhusi and Meck, 2005; Crystal, 2009), but some studies have suggested that intervals of several hours can be tracked (Pizzo and Crystal, 2006). According to the classical pacemaker accumulator theory, the circadian system may be implicated in interval timing as well. However, recent advances have challenged this view (Staddon and Higa, 1999; Wearden, 2004; Buhusi and Meck, 2005; Yin and Troger, 2011). Interval timing has been shown to be independent of the SCN and *Cry1,2* clock-genes (Lewis et al., 2003; Papachristos et al., 2011) and thus seems to be independent of the circadian system. To rule out the possibility that animals use this strategy for TPL, any distinctive external cues should be ruled out from experimentation, for instance by testing under constant light conditions to eliminate LD transitions. Previous test sessions may also start/stop/reset interval timing (intersession interval timing). Together with the ordinal strategy, this possibility can easily be ruled out by performing session skips.

Only when the above non-circadian strategies have been experimentally ruled out, one can conclude that animals must use a circadian timing strategy. In this case TPL is based on an internal clock, independent of external cues. The endogenous nature of cTPL can further be shown by identifying known oscillator characteristics, like persistence in constant conditions, and known limitations imposed by the circadian system (Crystal, 2009).

### Time memory: circadian retention paradigms vs. cTPL paradigms

Evidence that animals remember time of day is provided by behavioral paradigms that involve a training (stimulus encounter) followed by a retention test. In such experiments, animals show optimal retention when training and testing times match, indicating memory for the time of training. A basis for this knowledge was provided by Kamin who reported retention in a passive avoidance paradigm to be minimal one hour after training compared to the retention after 24 h, or even 19 days after training (Kamin, 1957). Why retention would be enhanced after a long interval compared to after a short interval was difficult to explain and the phenomenon, still known as the Kamin effect, has long been misinterpreted as a weak transition point in the processing of short-term memory into long term-memory. In 1973, Holloway and Wansley discovered that the Kamin effect was actually the result of a circadian periodicity in memory retention. They showed that, independent of the time of day at which training occurs, retention is always optimal 24 h later, or multiples thereof (Holloway and Wansley, 1973a,b; Wansley and Holloway, 1976). Next to passive avoidance, such periodic retention effects were also shown for active avoidance (Holloway and Wansley, 1973b; Cain et al., 2008), appetitive motivated learning (Wansley and Holloway, 1975), fear conditioning (Chaudhury and Colwell, 2002), conditioned place preference (Ralph et al., 2002; Valentinuzzi et al., 2008), and conditioned place avoidance (Cain et al., 2004a). However, it should be noted that not all mammals show this pattern (Oklejewicz et al., 2001).

The retention paradigms described above do not require animals to remember the time of training. Apparently animals remember time of day automatically. Therefore, Gallistel proposed that animals automatically encode time, together with place and the nature of biological significant events (Gallistel, 1990).

Although periodic retention deficits suggest time-memory, they do not necessarily implicate that animals learn an association between a stimulus and circadian phase. An alternative interpretation is that behavioral output (e.g., freezing) has been entrained by the stimulus pulse in the same way circadian rhythms can be entrained by other zeitgebers, such as LD transitions. However, when an animal is trained to go to different places at different times of day, as in TPL paradigms, more than entrainment of an oscillator must be involved: an association between time and place must have been learned (Biebach, 1989). In cTPL paradigms, memory of time is displayed directly by active choices. Animals are stimulated to remember the time because time discriminates between correct and incorrect choices. The correctness of a choice depends on correct memory and retention of time, based on previous encounters.

In summary, animals may use different strategies for TPL. The use of a circadian strategy is of special interest. cTPL presumes that an internal clock, and time derived from it, can be used by higher cognitive brain systems in adaptive experience based behavior. Correct location choices in cTPL implicate knowledge of current time of day (internal clock consultation), training times being stored as a contextual cue in memory (time-stamping, time-memory) and an association of these contextual time-points with spatial features and the nature of the event to guide behavior (decision making). These features make cTPL a unique circadian system dependent learning and memory ability and a tool to study the circadian system on a functional behavioral level. Next to providing a an up-to-date literature overview on TPL, this review will discuss the perspectives and functional aspects of (c)TPL.

## A literature overview on time-place learning

### From insects to birds to mammals

First TPL experiments were conducted studying honeybees. It was found that honeybees can be trained to collect food at any time of the day (Wahl, 1932), at multiple times (Koltermann, 1974), and at different places at different times (Finke, 1958). This plasticity implies that honeybees have a circadian oscillator at their disposal that allows for a continuous monitoring of the passage of time (Pittendrigh et al., 1958). Indeed, foraging behavior in bees was found to show underlying oscillator characteristics, like phase shifts, entrainment and limits of entrainment (Renner, 1959; Beier, 1968; Frisch and Aschoff, 1987), thus this internal oscillator could underlie TPL. Moore et al. showed evidence for cTPL in honeybees under laboratory conditions, thereby excluding a number of potential environmental cues which may have allowed for a contextual cue strategy, like circadian temperature-, humidity-, and light intensity fluctuations and position of the sun (Moore et al., 1989). But TPL in single, individually kept bees was never shown and also the use of ordinal- or interval timing strategies has never been ruled out in order to conclude cTPL (i.e., based on an internal clock).

Convincing evidence for cTPL came from experiments in birds. Field studies had already demonstrated anticipatory behavior regarding feeding schedules in kestrels (*Falco tinnunculus*). Individual kestrels showed an increased probability to hunt in the same area 24 h after having caught prey (Rijnsdorp et al., 1981; Wilkie et al., 1996). In a laboratory setting, Biebach and coworkers individually kept garden warblers (*Sylvia borin*) in a cage with four connected feeding rooms. The 12 h light phase was divided into four 3-h episodes in which entry to one of the feeding rooms was rewarded by food. Following an entry, all rooms were closed for 5 min, trapping the birds in the chosen location. This served as a punishment for wrong, non-rewarding location choices. Within ten days, the garden warblers made about 75% right (rewarding) choices. This pattern persisted when all rooms were rewarded and kept open, indicating time-place associated memory (Biebach, 1989). Conclusive evidence for cTPL was provided by Wenger and coworkers using starlings in a similar setup: in constant light conditions, the learned time and place associated feeding pattern was shown to free-run for almost a week, showing an important underlying oscillator property (Wenger et al., 1991).

Since Garden Warblers and starlings are both insectivorous birds, it was questioned whether the cTPL shown in these birds is specific for species that depend on a food source that shows daily fluctuations in availability. To test this idea, Falk investigated TPL in two related weaver bird species: an insectivore (*Ploceus bicolor*) and a granivore or seed-eater (*Euplectes hordeaceus*). Although the granivorous birds learned faster, they showed disrupted performance both after a session skip (all doors kept closed) and after a 6 h LD advance, suggesting that these birds used a non-cTPL strategy. In contrast, location visiting patterns of the insectivorous birds were unaffected by the session skip. After the 6 h LD advance these birds showed a phase advance in their feeding pattern that was less than 6 h, consistent with expectations based on an underlying circadian oscillator. In conclusion, these results suggest that not all species may have evolved cTPL ability.

TPL in mammals was first demonstrated using Long Evans rats, showing that rats can anticipate food at the correct location when it is made available at two different locations depending on the time of day (Boulos and Logothetis, 1990). It was known that SCN lesions, known to abolish LD entrainment and free-running circadian rhythms, did not interfere with food anticipatory rhythms or with the persistence of food anticipation during food deprivation (for review, see Boulos and Terman, 1980). Therefore, TPL was also investigated in SCN lesioned rats. These rats were able to master the task and this provided first evidence that TPL, like food anticipation, may be independent of the SCN (Boulos and Logothetis, 1990). Although the used strategy was not investigated, these findings were later confirmed by Mistlberger using male Wistar rats in a T-maze that were food deprived to 85–90% of *ad libitum* feeding weight. SCN intact- and SCN lesioned animals performed equally well. Moreover, session skips and an LD shift (day-night inversion) indicated that both groups used a circadian strategy. The authors suggested that an alternative internal oscillator, presumably a food entrainable oscillator (FEO), was used as a time source (Mistlberger et al., 1996).

Others had difficulties to show cTPL in rats. Carr and coworkers repeatedly found that Long Evans rats used an ordinal timing strategy, even when they tried to make an ordinal strategy unreliable by structural random session skips. They hypothesized that animals tested in a two sessions and two locations TPL setup may be more prone to adapt an ordinal strategy, because animals can simply alternate between locations (or learn to avoid always the previous rewarding location). Nonetheless, Long Evans rats also showed ordinal TPL in a three sessions and three locations TPL setup (Carr and Wilkie, 1997, 1999; Carr et al., 1999). Thorpe and coworkers also reported ordinal timing in this strain (Thorpe et al., 2003). Surprisingly, in multiple paradigms, Long Evans rats have also been reported not to show circadian retention deficits (McDonald et al., 2002; Cain et al., 2004c), indicating that this may be a strain specific deficit for time memory. On the other hand, Pizzo and coworkers found that Long Evans rats used primarily an interval timing strategy, but with evidence for a partial circadian strategy (Pizzo and Crystal, 2002). This indicates that these rats may not be unable to use a circadian strategy, but may less readily- or have more difficulty to do so.

### Decisive factors for TPL

Lukoyanov and coworkers underlined the role of food deprivation in TPL. They investigated whether Wistar rats could show TPL in a non-food reinforced time-place task. They used a Morris water maze in which the platform changed location between morning and afternoon sessions. Surprisingly, rats only learned the task when they were food deprived, receiving 60% of the daily *ad libitum* food consumption. *Ad libitum* fed animals and animals that were only food deprived to receive 90% of daily *ad libitum* food consumption did not perform above chance level. Although the use of an ordinal- or interval timing strategy was not excluded experimentally, the authors suggested that food deprivation enables access to the necessary temporal information from the FEO (Lukoyanov et al., 2002).

Widman and colleagues broadened this view. They had shown earlier that an increased response cost can trigger TPL. Female Sprague Dawley rats showed TPL when they had to climb for food in a vertical maze, while rats tested in a horizontal maze did not. Moreover, the number of rats showing TPL increased as the height was increased (Widman et al., 2000). However, in this experiment the rats were still food deprived to 80% of *ad libitum* feeding weight. In a following experiment, the group of Widman used *ad libitum* fed male Sprague Dawley rats in a Morris water maze. Again, without food deprivation or an increased response cost, the rats did not show TPL, replicating the earlier findings of Lukoyanov et al. (2002). A second group of rats was also tested under *ad libitum* feeding conditions, but this time the response cost was increased by adding a weight belt to the rats. These rats did show TPL. Although the authors did not exclude the possibility that animals used an ordinal or interval timing strategy to solve the task, the authors argument that activation of a food system (FEO) is not specifically necessary for TPL. The authors suggested that food deprivation also forms an increased response cost (relative effort). The authors hypothesized that either the FEO or the SCN can be used as a consulted clock for TPL, and that access to-, or activation of these systems is facilitated by an increased response cost (Widman et al., 2004). In line with this, Aragona and coworkers showed that TPL could be facilitated in a two sessions and two locations lever pressing study, simply by placing a water bottle in between the levers (Aragona et al., 2002). This may also be explained by the response cost hypothesis proposed by Widman et al. because animals now had to walk around the water bottle if they had initially chosen a wrong (non-rewarding) lever (location). Thus, a high response cost seems to facilitate TPL, presumably because it stimulates animals to not make incorrect decisions and thus the need or motivation to encode the time-place contingency.

TPL has however also been demonstrated in experimental setups involving a low response cost and without food deprivation, using a palatable food resource (Means et al., 2000; Thorpe and Wilkie, 2007). This suggests that a low response cost may be partly compensated by an increased reward value. From another perspective, increasing the reward value (by using a palatable food reward or more severe food deprivation) may also indirectly increase the response cost, because the satisfaction of a subjectively more intense craving is delayed when animals have initially chosen a non-rewarding location and have to switch between locations.

Taken together, previous TPL studies have identified two critical factors to induce TPL: A reward to induce goal directed behavior and a response cost to stimulate animals to make correct choices. Both of these factors seem to add to the significance or motivational value for an animal to encode the time-place contingency. Recently, these principles have been implemented in a novel TPL paradigm for mice (Van der Zee et al., 2008). This paradigm will be described in the following section along with recently obtained results using this setup.

## A novel TPL test for mice

**Figure 1:**
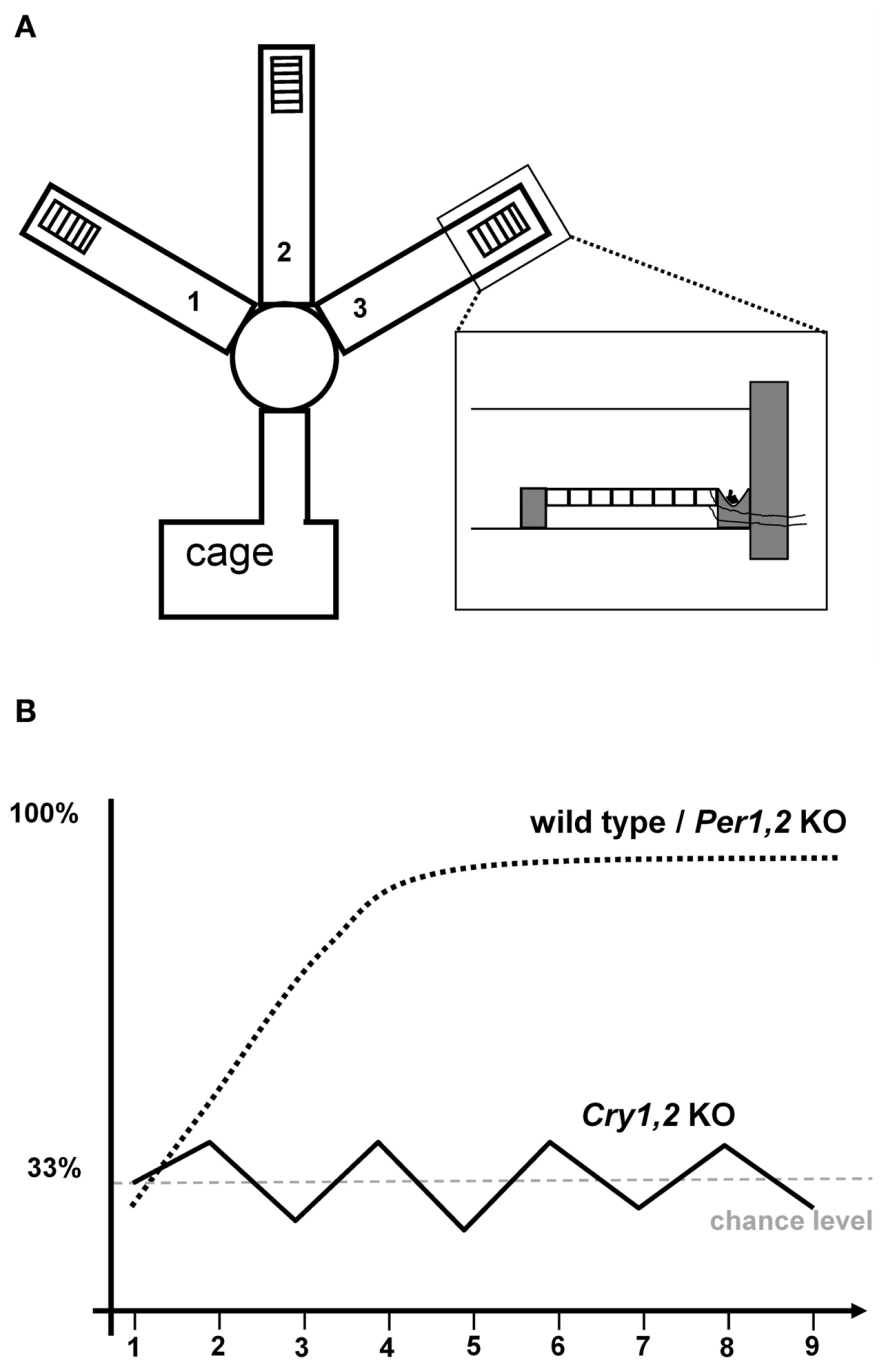
**Schematic drawing of the TPL maze used by Van der Zee et al. (2008).** Food is placed behind a metal grid on which mice have to stand to reach it, providing the possibility to administer a mild foot shock **(A)**. Schematic representations of the learning curve: wild-type and *Per1,2* mutant mice readily learn to avoid the “time-of-day dependent” negatively reinforced location. In contrast, *Cry1,2* knockout mice cannot master the task and remain at the 33% chance level **(B)**.

Despite the extended literature on TPL in rats, albeit with differential conclusions, the lack of studies in mice was surprising and perhaps partly based on the absence of a suitable paradigm. This inspired our group to design a novel TPL paradigm for mice. The rational was to induce a conflict between a positive reinforcer (food reward) and a negative reinforcer depending on the time of day. The latter was implemented by applying a mild foot shock through a grid located in front of the food reward (see Figure 1A). Mice had to step on the grid to be able to acquire the food reward. To motivate the mice to search for food, they were food deprived to 85% of their *ad libitum* body weight. As such, the paradigm emulates the natural situation in which hungry animals seek food while different feeding locations can be predictably safe or unsafe to visit depending on the time of day. We decided to use three times of day (sessions) in a three-arm maze. For example, a mouse was tested at 9:00, 12:00, and 15:00 o'clock. In each of the three daily test sessions, mice had to learn to avoid one of the three baited arms, i.e., the one in which they would receive the mild foot shock if trying to reach the food reward. For example, at 9:00 this was in the left arm, at 12:00 in the middle arm, and at 15:00 in the right arm. A session was performed correctly if the arm with the foot shock was visited last or fully avoided after first having visited both correct locations. After habituation steps, wild-type C57Bl6 mice readily learned this task, reaching an average performance of approximately 80% correct choices in just five days (Figure 1B). Moreover, session skips (to identify the use of an ordinal strategy) and testing under a constant light condition (to identify the use of an interval timing strategy) revealed that the animals were using a circadian strategy (Van der Zee et al., 2008).

To our knowledge, this was the first time cTPL was shown in mice. This has opened new possibilities to examine the neuronal substrates underlying cTPL, as well as presumably involved clock genes. Various mice, knockout for specific clock genes, can now be tested behaviorally for the functional involvement of these genes in a time-of-day dependent memory test. In following sections, we will review the first published results on *Cry1* and *Cry2* knockout mice. In addition, we report on preliminary results of a follow up study in *Per1* and *Per2* knockout mice.

### TPL in *Cry* and *Per* mutant mice

cTPL is a unique way to study clock-genes on a functional, behavioral level. We introduced clock-gene deficient mice in the context of TPL, and showed that young *Cry*1^−/−^*Cry*2^−/−^ (referred to as *Cry1,2*) double knockout male C57Bl6 mice were unable to master the paradigm (Van der Zee et al., 2008). Performance stayed around chance level (Figure 1B). Wild-type mice included in this experiment (of same sex, age, and strain) successfully mastered the paradigm (Figure 1B). Session skips under either LD or LL conditions did not affect performance of wild-type mice, replicating that wild-type mice use a circadian strategy in the used setup.

This finding, that cTPL is *Cry* dependent, confirmed the circadian nature of cTPL and the functional role of Cry genes in circadian rhythms. In the context of this review, these results however raise a relevant question. Other studies have shown that animals can use alternative non-circadian strategies to solve a daily TPL task. Given that *Cry1,2* deficient mice have a distorted internal clock thus raises the question why these mice did not use a non-circadian strategy, like an ordinal- or interval timing strategy, to master the paradigm. The possibility that these animals had a general learning deficit was ruled out by control experiments that showed intact spatial memory and association abilities (contextual fear conditioning and spatial learning in a Y maze; Van der Zee et al., 2008). Clearly the used setup initially induces the circadian strategy as seen in wild-type mice. Nevertheless, feedback from the testing (repeated mistakes leading to foot-shocks) theoretically should have led animals to abandon this unreliable strategy for one that produces better outcome. Several reasons may explain why this did not occur. First, animals may only abandon the circadian strategy if the clock signal gets too weak, as with aging, but not when the memory system gets a clear (but distorted) signal as may be the case with young *Cry1,2* knockout mice. Second, session skips may have led animals to mark the ordinal strategy as unreliable as well, resulting in sticking with the circadian strategy. Indeed sessions were already skipped during habituation and on the third day of actual testing. Third, total test duration may have been too short. If the testing would have continued for a longer time (without session skips), the *Cry1,2* knockout mice may have switched to a non-circadian strategy. Finally, although interval timing has been shown to be independent of *Cry* genes (Papachristos et al., 2011) ordinal timing may not be. Future studies should confirm if *Cry1,2* double knockout mice can learn TPL using a non-circadian strategy. Nonetheless, the results demonstrated that the time-of-day signal as used in our TPL paradigm must originate from an underlying CRY dependent (circadian) mechanism.

The finding that *Cry1,2* knockout mice could not learn TPL, raises the question whether this inability is *Cry* gene specific, or that (c)TPL depends on other clock genes as well. Using the exact same setup and methods as with the *Cry1,2* knockout mice, here we report on some new findings investigating TPL in *Per1*^−/−^*Per2*^−/−^ (referred to as *Per1,2*) double mutant mice (Zheng et al., 1999). We found that *Per1,2* mutant mice can learn TPL indistinguishable from wild-type mice (Figure 1B). Hippocampus dependent contextual and spatial learning has been shown to be unaffected in *Per1,2* mutant mice (Zueger et al., 2006), indicating that such factors would indeed not prevent these mutant mice to show TPL by using a non-circadian (ordinal-) strategy. However, session skips and prolonged testing under constant light conditions had no effect on performance, indicating that these mice used a circadian strategy (cTPL). This was unexpected from a genotype that is known to lead to arrhythmicity on both a behavioral and molecular level and raises new questions on the role of *Per* genes in circadian behavior and memory (Mulder et al., 2011; Mulder et al., submitted).

## Perspectives and functional aspects of time-place learning

TPL provides animals with an adaptive mechanism to guide spatiotemporal behavior in accordance with prior experience. cTPL implies that distinct phases of an internal circadian rhythm can participate in associative memory. Studying this type of learning and memory has potentials for several fields of cognitive neuroscience. Below we will outlay some of the perspectives and functional aspects of (c)TPL.

### Memory

(c)TPL may demonstrate the formation of tripartite memory association codes. Gallistel stated that whenever an animal encounters a biological significant event, it automatically creates a tripartite memory code consisting of the nature of the event, when the event occurred and where the event occurred (Gallistel, 1990; Wilkie, 1995). The historical difficulties with demonstrating TPL in rats have raised doubts regarding this theory. A study by Thorpe et al. suggests that instead of automatically coding tripartite memory codes on the encounter of important events, animals may automatically create bipartite memory codes, i.e., time-event (and event-place under some circumstances). Thorpe et al. hypothesize that these bipartite codes may later be transformed into tripartite memory codes, but only under high response cost conditions (Thorpe and Wilkie, 2007). Indeed, rats readily show time-of-day discriminations using go/no-go designs in which the value of the reward differs between sessions, without a distinction between different locations (Means et al., 2000; Thorpe et al., 2003). In such designs, animals demonstrate time memory by showing a shorter reaction time at session(s) with a higher reward value. Because a distinction between locations is not necessary in such setups, only bipartite memory codes (time-event) are required, while TPL theoretically requires tripartite memory codes and thus a high response cost (Thorpe and Wilkie, 2007). Future TPL studies may shed more light on the organization of associative memory.

### Conditional use of the circadian strategy

The distinction between TPL and cTPL is not always apparent in the literature, because the used strategy is not always investigated. Therefore, little is known on the decisive factors for the utilization of particular strategies. Differential conclusions on the used strategy for TPL within the same strain of specie, indicate that the adapted strategy may not only depend on the used organism or strain, as was concluded from a comparison between seed- and insect eating bird species (Falk, 1992), but on other factors as well. For instance, while cTPL was shown in Wistar rats (Mistlberger et al., 1996), Pizzo et al. reported the utilization of an ordinal strategy by this strain. The authors argument that, although rats may have information on time of day available, they do not readily use this information when other strategies are available, and that, when only two locations and two times have to be distinguished, ordinal timing may be the easiest solution to adapt (Pizzo and Crystal, 2004). However, Mistlberger et al. (1996) had also used a two sessions and two locations TPL setup, indicating that other operational factors must play a role in which strategy animals use for TPL. Theoretically, subjective reward- and response cost values may not only determine whether animals show TPL, but also influence the used strategy. When the stakes are high, memory should be optimal and fault proof. The circadian strategy is theoretically the most reliable strategy because it does not depend on external cues which can be unreliable or remain unnoticed. For instance, with an ordinal strategy, a missed feeding opportunity (similar to a session skip) would result in visiting all following locations at the wrong time of day, which may result in less or no resource collection, increased energy expenditure and/or higher risk of encountering a predator. In expense, a circadian strategy, potentially including the formation of tripartite memory codes, may be more costly on brain resources to create and/or maintain, and may therefore only be recruited when “the stakes” are relatively high. As Falk already stated, perhaps the granivorous weaver birds, showing non-cTPL, learned faster because they learned less (Falk, 1992). From another perspective, it makes sense that the brain does not recruit circadian timekeeping mechanisms for insignificant events. In addition, subjective reward- and response cost value thresholds will likely differ between species and even individuals. This may account for some of the discrepancies found in the literature regarding whether animals showed TPL or not and regarding the used strategies. Thus, although speculative, higher reward- and response cost values may not only induce TPL (Widman et al., 2004) and the formation of tripartite memory codes (Thorpe and Wilkie, 2007), but also the use of a circadian strategy (cTPL). Future studies may confirm this hypothesis.

### Clockgenes

cTPL in mice offers a way to study the role of clock genes in a functional and behavioral paradigm (Van der Zee et al., 2008). cTPL was found dependent of *Cry1* and/or *Cry2* genes and encoded proteins. However, internal timekeeping underlying cTPL seems to be buffered against missing *Per1* and *Per2* clock genes, even though *Cry1,2* and *Per1,2* double mutant mice are both behaviorally arrhythmic when housed in absence of a (circadian) LD cycle. This provides new yet underexplored insights regarding the role of these clock genes in the circadian system and memory (Mulder et al., 2011; Mulder et al., submitted). The discrepancy in TPL ability between *Cry* and *Per* knockout mice indicates that TPL can be a discriminating paradigm to investigate functional behavioral functionality of known clock-genes and validates the investigation of other molecular clock components.

### Aging

**Figure 2:**
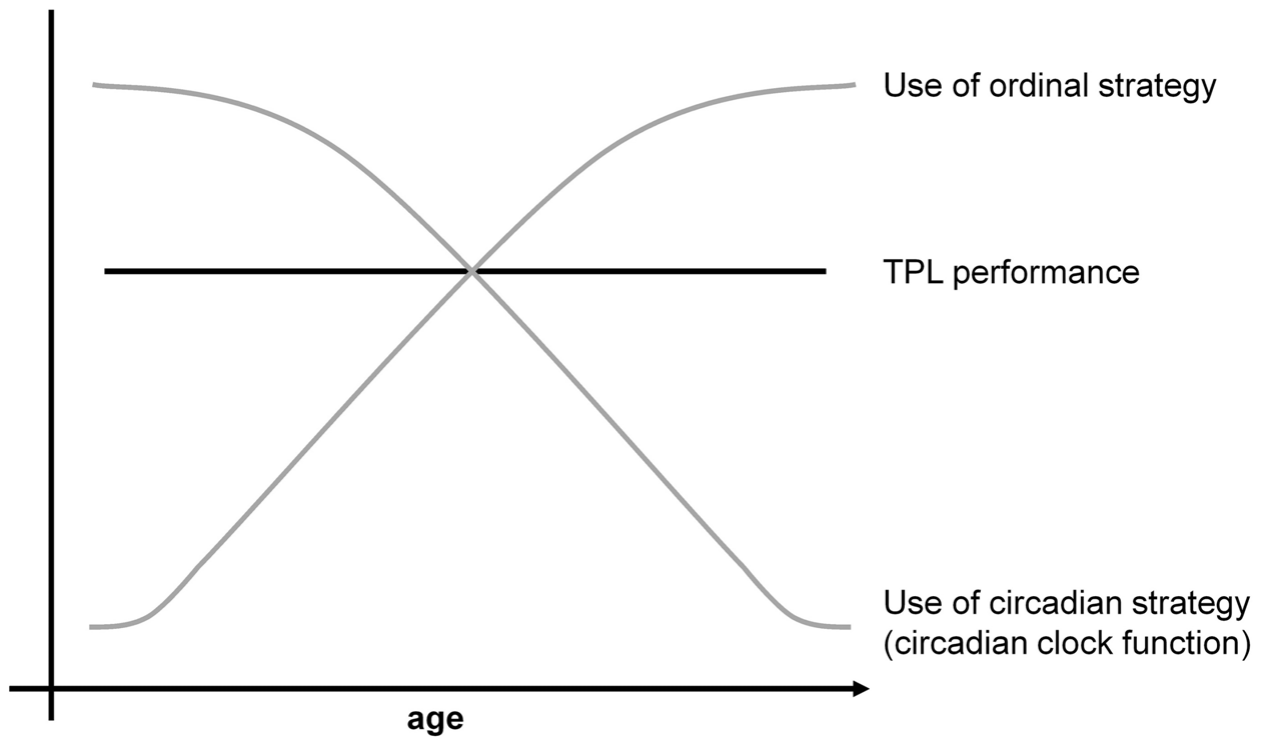
**Schematic representation of the strategy switching hypothesis with aging.** Circadian system decline predicts that the circadian strategy becomes increasingly unreliable with age. To buffer performance loss, the ordinal strategy may gain dominance with aging as a reference to guide TPL behavior.

TPL has potential as an animal model for episodic memory and aging. Such functional behavioral models are scarce, yet essential to test interventions that potentially improve detrimental effects of aging and (episodic) memory related diseases like Alzheimer's disease (AD). Patients suffering from AD are often said to be disorientated in time and place, and memories of when and where things happened (episodic memory) are among the first to be affected in AD patients. TPL research investigates the functional integration of what, when and where and may thus provide insight on the origin of these early onset AD symptoms (Dere et al., 2005a,b). Moreover, deterioration of the circadian system with aging has been related to other age-associated pathologies (Kondratova and Kondratov, 2012). Despite the potential, TPL has not been investigated before in the context of aging. Circadian system deterioration, such as seen with aging or otherwise, predicts that the cTPL strategy will become less reliable. To buffer performance loss, animals may show to switch the dominant strategy that guides their behavior to one that is independent of the circadian system. In a first investigation of TPL with aging, we found preliminary evidence for this *switching hypothesis* (Figure 2). Middle-aged (45 weeks old) and aged (80 weeks old) mice showed indistinguishable TPL performance. Session skipping revealed that aged mice primarily used an ordinal strategy while TPL performance of middle-aged mice was significantly less affected by session skips (Mulder et al., 2010; Mulder et al., submitted). Although these results are preliminary due to relatively low numbers of animals, these data suggest that cTPL is more age sensitive than TPL. Animals will first abandon the circadian strategy for one that is independent of the circadian system, before showing age related performance loss, adding aging as a factor for the conditional use of a specific TPL strategy.

The switching hypothesis supports a general theory of aging as a loss of behavioral and cognitive flexibility, in this case losing the ability to use the circadian strategy. To compensate performance loss, alternative strategies are explored. In humans, compensatory mechanisms have been demonstrated by the activation of more brain areas for a cognitive task compared to younger individuals (Logan et al., 2002). A similar phenomenon is seen in sleep deprivation. Aged mice and sleep deprived young mice perform just as well as non-sleep deprived young mice in Y-maze learning (a two-arm maze setting without fixed time-of-day testing), showing a similar learning curve. However, they show deficits in reversal learning (Hagewoud et al., 2010; Havekes et al., 2011). Studies have shown that sleep deprived mice rely more on an alternative strategy requiring the striatum (procedural memory) instead of the hippocampus, which is true for aged mice as well (Hagewoud et al., 2010). Although speculative, the striatum may not harbor the necessary connectivity with the circadian system. Future studies will have to confirm the switching hypothesis and may further validate TPL as a model for episodic memory.

cTPL presumes a connection between the circadian system and memory, but not much is known about this connection regarding the origin of the underlying circadian oscillator and the molecular/neuronal signaling to the memory system. cTPL provides the means to investigate this connection. This will be the focus of the final section of this review.

## Putative mechanism underlying cTPL

The internal circadian process underlying cTPL currently remains elusive. The SCN (central pacemaker) would be a first educated guess. Via neuropeptidergic, nonsynaptic pathways the SCN is connected to brain regions involved in learning and memory (Van der Zee et al., 2009 and references therein). One of the major output systems of the SCN is the neuropeptidergic Vasopressin (AVP) system. AVP released in the third ventricle reaches numerous brain regions involved in learning and memory, including the hippocampus. AVP receptors are expressed in the hippocampus, and AVP is able to induce LTP in various sub-regions of the hippocampus (Dubrovsky et al., 2003). The AVP neurons of the SCN and the formation of time memory are regulated by the cholinergic input (Hut and Van der Zee, 2011), by which the SCN may provide a time cue to support the formation of time-place associations in the hippocampus. Moreover, both the AVP system (Van der Zee et al., 1999) and cholinergic signaling in the SCN (Van der Zee et al., 1991) are strikingly age-sensitive. Aged rats show age-specific alteration in the AVP and cholinoceptive systems in the SCN (Biemans et al., 2003), predicting age-related impairments in cTPL performance. Although the SCN is known to be arrhythmic in *Cry1,2* and *Per1,2* double mutant mice, it has been found that the SCN in *Per1,2* mutant mice retains some functionality (van der Veen et al., 2008), which may explain why *Per1,2* mutant mice still showed cTPL. Taken together, the SCN seems a good candidate as the circadian clock underlying cTPL. However, SCN lesioned rats were still able to show cTPL (Mistlberger et al., 1996), indicating that the SCN is not a prerequisite for cTPL. On the other hand, we found that a 3 h phase shift induced by a light pulse affects feeding patterns in our TPL paradigm (unpublished data), suggesting at least partial or modulator involvement of the SCN.

An alternative candidate for circadian regulation of TPL is the FEO (Stephan, 2002). Despite many lesion studies this oscillator has not yet been fully localized (Davidson, 2006). Currently the FEO is thought to be comprised of a network of interconnected brain structures (Carneiro and Araujo, 2009). cTPL has also been shown in experiments that did not involve food (Widman et al., 2004), arguing against the FEO as the timing mechanism behind cTPL. Another identified oscillator is the methamphetamine sensitive circadian oscillator (MASCO) (Hiroshige et al., 2009), but it has been hypothesized that both the FEO and the MASCO may be a manifestation of the same oscillator induced by arousal (Cain and Ralph, 2009). Together, these known oscillators may drive the circadian expression of many potential time cues for cTPL.

Several criteria may narrow the search: (1) Potential candidate cues should obey the characteristic of being expressed in a circadian fashion. (2) As the hippocampus is the key region for learning and spatial memory, the cue should be able to reach the hippocampus through either synaptic or hormonal routes. (3) In turn the hippocampus should be receptive for the cue. Given these criteria, next to the already discussed SCN derived AVP, potential candidates may be hormones like leptin, ghrehlin, corticosteron, glucagon, and insulin (Lukoyanov et al., 2002; Carneiro and Araujo, 2009), or neurotransmitters like dopamine (Aragona et al., 2002). In addition, non-hormonal metabolic signals, like free fatty acids, ketone bodies and glucose could function as a food driven hourglass timer, for instance, through depletion of glycogen stores or through SIRT1, an NAD(+)-dependent deacetylase which is known to modulate CLOCK-BMAL1 activity (Asher et al., 2008). In addition, multiple time signals and underlying oscillators and/or metabolic hourglass mechanisms may influence subjective timing, as for instance seen in the regulation of sleep (Daan et al., 1984).

Recent studies have shown that the hippocampus harbors its own clockwork. Clock genes are expressed in most regions of the hippocampus (CA1, CA3, DG) and peak expression of some clock genes seems to coincide with times at which memory is consolidated (Feillet et al., 2008; Jilg et al., 2010). Moreover, memory deficits have been shown in clock gene knockout animals (Sakai et al., 2004; Lyons et al., 2006; Feillet et al., 2008; Jilg et al., 2010). Recently, memory formation and consolidation were shown to depend on the circadian reactivation of the cAMP/MAPK/CREB pathway in hippocampal neurons (Eckel-Mahan et al., 2008). Although the circadian expression of clock genes (*Per2*) has been shown in isolated hippocampal slices (Wang et al., 2009), MAPK cycling seems to be driven by the SCN (Phan et al., 2011). In the SCN, activation of the MAPK pathway is light responsive (Obrietan et al., 1998; Butcher et al., 2002). It has been hypothesized that training might function similarly in hippocampal neurons as light activation and clock resetting functions in SCN neurons; inducing a unique, temporally specific molecular profile in memory-specific neuronal ensembles (Gerstner et al., 2009). Indeed, BMAL1 is negatively regulated by MAPK (Sanada et al., 2002), providing a molecular clock resetting mechanism by training. Interestingly, next to “place” cells in the hippocampal CA1 and CA3 region, “time” cells have recently been identified and shown to track the elapsing of an interval (Macdonald et al., 2011; Shapiro, 2011). It is likely that this hippocampal clock may not only be entrained by training (event encounters), but is also modulated by internal cues like SCN and FEO outputs.

In conclusion, TPL offers research potentials in the neurobiological framework of circadian clock regulation, memory function, episodic-like-memory, and aging. A clear next step is to clarify the neurobiological mechanisms underlying cTPL.

### Conflict of interest statement

The authors declare that the research was conducted in the absence of any commercial or financial relationships that could be construed as a potential conflict of interest.

## References

[B1] Antoniadis E. A. Ko C. H. Ralph M. R. McDonald R. J. (2000). Circadian rhythms, aging and memory. Behav. Brain Res. 114, 221–233 10.1016/S0166-4328(00)00290-4 10996063

[B2] Aragona B. J. Curtis J. T. Davidson A. J. Wang Z. Stephan F. K. (2002). Behavioral and neurochemical investigation of circadian time-place learning in the rat. J. Biol. Rhythms 17, 330–344 10.1177/074873002129002636 12164249

[B3] Aschoff J. (1954). Zeitgeber der tierischen Tagesperiodik. Naturwissenschaften 41, 49–56

[B4] Asher G. Gatfield D. Stratmann M. Reinke H. Dibner C. Kreppel F. (2008). SIRT1 Regulates Circadian Clock Gene Expression through PER2 Deacetylation. Cell 134, 317–328 10.1016/j.cell.2008.06.050 18662546

[B5] Beier W. (1968). Beeinflussung der inneren Uhr der Bienen durch Phasenverschiebung des Licht-Dunkel-Zeitgebers. Z. Bienenforsch. 9, 356–378

[B6] Beling I. (1929). Über das Zeitgedächtnis der Bienen. Z. Vergl. Physiol. 9, 259–338

[B7] Biebach H. (1989). Time-and-place learning by garden warblers, *Sylvia-Borin*. Anim. Behav. 37, 353–360

[B8] Biemans B. A. Van der Zee E. A. Daan S. (2003). Age-dependent effects of conditioning on cholinergic and vasopressin systems in the rat suprachiasmatic nucleus. Biol. Chem. 384, 729–736 10.1515/BC.2003.081 12817469

[B9] Boulos Z. Logothetis D. E. (1990). Rats anticipate and discriminate between two daily feeding times. Physiol. Behav. 48, 523–529 10.1016/0031-9384(90)90294-E 2075203

[B10] Boulos Z. Terman M. (1980). Food availability and daily biological rhythms. Neurosci. Biobehav. Rev. 4, 119–131 6106914 10.1016/0149-7634(80)90010-x

[B11] Budzynski C. A. Dyer F. C. Bingman V. P. (2000). Partial experience with the arc of the sun is sufficient for all-day sun compass orientation in homing pigeons, *Columba livia*. J. Exp. Biol. 203, 2341–2348 10887072 10.1242/jeb.203.15.2341

[B12] Buhusi C. V. Meck W. H. (2005). What makes us tick? Functional and neural mechanisms of interval timing. Nat. Rev. Neurosci. 6, 755–765 10.1038/nrn1764 16163383

[B13] Butcher G. Q. Doner J. Dziema H. Collamore M. Burgoon P. W. Obrietan K. (2002). The p42/44 mitogen-activated protein kinase pathway couples photic input to circadian clock entrainment. J. Biol. Chem. 277, 29519–29525 10.1074/jbc.M203301200 12042309

[B14] Cain S. W. Chou T. Ralph M. R. (2004a). Circadian modulation of performance on an aversion-based place learning task in hamsters. Behav. Brain Res. 150, 201–205 10.1016/j.bbr.2003.07.001 15033293

[B15] Cain S. W. Karatsoreos I. Gautam N. Konar Y. Funk D. McDonald R. J. (2004b). Blunted cortisol rhythm is associated with learning impairment in aged hamsters. Physiol. Behav. 82, 339–344 10.1016/j.physbeh.2004.04.004 15276797

[B16] Cain S. W. Ko C. H. Chalmers J. A. Ralph M. R. (2004c). Time of day modulation of conditioned place preference in rats depends on the strain of rat used. Neurobiol. Learn. Mem. 81, 217–220 10.1016/j.nlm.2004.02.003 15082023

[B17] Cain S. W. McDonald R. J. Ralph M. R. (2008). Time stamp in conditioned place avoidance can be set to different circadian phases. Neurobiol. Learn. Mem. 89, 591–594 10.1016/j.nlm.2007.07.011 17905603

[B18] Cain S. W. Ralph M. R. (2009). Circadian modulation of conditioned place avoidance in hamsters does not require the suprachiasmatic nucleus. Neurobiol. Learn. Mem. 91, 81–84 10.1016/j.nlm.2008.10.005 19013252

[B19] Carneiro B. T. Araujo J. F. (2009). The food-entrainable oscillator: a network of interconnected brain structures entrained by humoral signals? Chronobiol. Int. 26, 1273–1289 10.3109/07420520903404480 19916831

[B20] Carr J. A. R. Tan A. O. Wilkie D. M. (1999). Further evidence that rats use ordinal timing in a daily time–place learning task. Behav. Processes 48, 35–48 10.1016/s0376-6357(99)00074-1 24897561

[B21] Carr J. A. R. Wilkie D. M. (1997). Rats use an ordinal timer in a daily time-place learning task. J. Exp. Psychol. Anim. Behav. Process. 23, 232–247 10.1037/0097-7403.23.2.232 9095544

[B22] Carr J. A. R. Wilkie D. M. (1999). Rats are reluctant to use circadian timing in a daily time–place task. Behav. Processes 44, 287–299 10.1016/s0376-6357(98)00036-9 24897230

[B23] Carrier J. Monk T. H. (2000). Circadian rhythms of performance: new trends. Chronobiol. Int. 17, 719–732 11128289 10.1081/cbi-100102108

[B24] Chaudhury D. Colwell C. S. (2002). Circadian modulation of learning and memory in fear-conditioned mice. Behav. Brain Res. 133, 95–108 10.1016/S0166-4328(01)00471-5 12048177

[B25] Craig L. A. McDonald R. J. (2008). Chronic disruption of circadian rhythms impairs hippocampal memory in the rat. Brain Res. Bull. 76, 141–151 10.1016/j.brainresbull.2008.02.013 18395623

[B26] Crystal J. D. (2009). Theoretical and conceptual issues in time-place discrimination. Eur. J. Neurosci. 30, 1756–1766 10.1111/j.1460-9568.2009.06968.x 19863655 PMC2783770

[B27] Daan S. Beersma D. G. Borbely A. A. (1984). Timing of human sleep: recovery process gated by a circadian pacemaker. Am. J. Physiol. 246, R161–R183 6696142 10.1152/ajpregu.1984.246.2.R161

[B28] Daan S. Koene P. (1981). On the timing of foraging flights by oystercatchers, haematopus ostralegus, on tidal mudflats. Neth. J. Sea Res. 15, 1–22

[B29] Davidson A. J. (2006). Search for the feeding-entrainable circadian oscillator: a complex proposition. Am. J. Physiol. Regul. Integr. Comp. Physiol. 290, R1524–R1526 10.1152/ajpregu.00073.2006 16455773

[B30] Dere E. Huston J. P. Souza Silva M. A. (2005a). Episodic-like memory in mice: simultaneous assessment of object, place and temporal order memory. Brain Res. Brain Res. Protoc. 16, 10–19 10.1016/j.brainresprot.2005.08.001 16185914

[B31] Dere E. Huston J. P. Souza Silva M. A. (2005b). Integrated memory for objects, places, and temporal order: evidence for episodic-like memory in mice. Neurobiol. Learn. Mem. 84, 214–221 10.1016/j.nlm.2005.07.002 16102980

[B32] Devan B. D. Goad E. H. Petri H. L. Antoniadis E. A. Hong N. S. Ko C. H. (2001). Circadian phase-shifted rats show normal acquisition but impaired long-term retention of place information in the water task. Neurobiol. Learn. Mem. 75, 51–62 10.1006/nlme.1999.3957 11124046

[B33] Dibner C. Schibler U. Albrecht U. (2010). The mammalian circadian timing system: organization and coordination of central and peripheral clocks. Annu. Rev. Physiol. 72, 517–549 10.1146/annurev-physiol-021909-135821 20148687

[B34] Dubrovsky B. Tatarinov A. Gijsbers K. Harris J. Tsiodras A. (2003). Effects of arginine-vasopressin (AVP) on long-term potentiation in intact anesthetized rats. Brain Res. Bull. 59, 467–472 10.1016/S0361-9230(02)00961-9 12576144

[B35] Eckel-Mahan K. L. Phan T. Han S. Wang H. Chan G. C. Scheiner Z. S. (2008). Circadian oscillation of hippocampal MAPK activity and cAmp: implications for memory persistence. Nat. Neurosci. 11, 1074–1082 19160506 10.1038/nn.2174 PMC2772165

[B36] Enright J. T. (1970). Ecological aspects of endogenous rhythmicity. Ann. Rev. Ecol. Systemat. 1, 221–238 10.1016/B978-0-12-387690-4.00004-0 21924976

[B37] Enright J. T. (1975). The circadian tape recorder and its entrainment, in Physiological Adaptation to the Environment, ed Vernberg F. J. (New York, NY: Intext Educational), 465–476

[B38] Falk H. (1992). Learning a time-place pattern of food availability. Behav. Ecol. Sociobiol. 31, 9–15 1773101

[B39] Feillet C. A. Mendoza J. Albrecht U. Pevet P. Challet E. (2008). Forebrain oscillators ticking with different clock hands. Mol. Cell. Neurosci. 37, 209–221 10.1016/j.mcn.2007.09.010 17996461

[B40] Fekete M. van Ree J. M. Niesink R. J. de W. D. (1985). Disrupting circadian rhythms in rats induces retrograde amnesia. Physiol. Behav. 34, 883–887 4059376 10.1016/0031-9384(85)90008-3

[B41] Finke I. (1958). Zeitgedächtnis und Sonnenorientierung der Bienen. Lehramtsarbeit Naturw Fak Univ Munchen, München

[B42] Folkard S. Hume K. I. Minors D. S. Waterhouse J. M. Watson F. L. (1985a). Independence of the circadian rhythm in alertness from the sleep/wake cycle. Nature 313, 678–679 3974700 10.1038/313678a0

[B43] Folkard S. Marks M. Minors D. S. Waterhouse J. M. (1985b). Circadian rhythms in human performance and affective state. Acta Psychiatr. Belg. 85, 568–581 4091019

[B44] Frisch B. Aschoff J. (1987). Circadian rhythms in honeybees: entrainment by feeding cycles. Physiol. Entomol. 12, 41–49

[B45] Frisch K. (1950). Die Sonne als Kompaß im Leben der Bienen. Cell. Mol. Life Sci. 6, 210–221 15421336 10.1007/BF02173654

[B46] Gallistel C. R. (1990). Representations in animal cognition: an introduction. Cognition 37, 1–22 10.1016/0010-0277(90)90016-D 2269003

[B47] Gerstner J. R. Lyons L. C. Wright K. P. Jr. Loh D. H. Rawashdeh O. Eckel-Mahan K. L. (2009). Cycling behavior and memory formation. J. Neurosci. 29, 12824–12830 10.1523/JNEUROSCI.3353-09.2009 19828795 PMC4077269

[B48] Gould J. L. (1987). Honey bees store learned flower-landing behaviour according to time of day. Anim. Behav. 35, 1579–1581

[B49] Hagewoud R. Havekes R. Tiba P. A. Novati A. Hogenelst K. Weinreder P. (2010). Coping with sleep deprivation: shifts in regional brain activity and learning strategy. Sleep 33, 1465–1473 21102988 10.1093/sleep/33.11.1465 PMC2954696

[B50] Harrison J. M. Breed M. D. (1987). Temporal learning in the giant tropical ant, Paraponera clavata. Physiol. Entomol. 12, 317–320

[B51] Havekes R. Abel T. Van der Zee E. A. (2011). The cholinergic system and neostriatal memory functions. Behav. Brain Res. 221, 412–423 10.1016/j.bbr.2010.11.047 21129408 PMC3075367

[B52] Hiroshige T. Honma K. Honma S. (2009). SCN-Independent circadian oscillators in the rat. Brain Res. Bull. 27, 441–445 1959043 10.1016/0361-9230(91)90139-b

[B53] Hoffmann K. (1960). Experimental manipulation of the orientational clock in birds. Cold Spring Harb. Symp. Quant. Biol. 25, 379–387 13715052 10.1101/sqb.1960.025.01.040

[B54] Holloway F. A. Wansley R. (1973a). Multiphasic retention deficits at periodic intervals after passive-avoidance learning. Science 180, 208–210 10.1126/science.180.4082.208 4694308

[B55] Holloway F. A. Wansley R. A. (1973b). Multiple retention deficits at periodic intervals after active and passive avoidance learning. Behav. Biol. 9, 1–14 4738709 10.1016/s0091-6773(73)80164-6

[B56] Hut R. A. Van der Zee E. A. (2011). The cholinergic system, circadian rhythmicity, and time memory. Behav. Brain Res. 221, 466–480 10.1016/j.bbr.2010.11.039 21115064

[B57] Jilg A. Lesny S. Peruzki N. Schwegler H. Selbach O. Dehghani F. (2010). Temporal dynamics of mouse hippocampal clock gene expression support memory processing. Hippocampus 20, 377–388 10.1002/hipo.20637 19437502

[B58] Kamin L. J. (1957). The retention of an incompletely learned avoidance response. J. Comp. Physiol. Psychol. 50, 457–460 13481182 10.1037/h0044226

[B59] Keeton W. T. (1974). The mystery of pigeon homing. Sci. Am. 231, 96–97 4439005 10.1038/scientificamerican1274-96

[B60] Koltermann R. (1974). Periodicity in the activity and learning performance of the honey bee, in Experimental Analysis of Insect Behaviour, ed Browne B. L. (Heidelberg, Berlin: Springer-Verlag), 218–227

[B61] Kondratova A. A. Kondratov R. V. (2012). The circadian clock and pathology of the ageing brain. Nat. Rev. Neurosci. 13, 325 10.1038/nrn3208 22395806 PMC3718301

[B62] Kramer G. (1950). Weitere analyse der faktoren, welche die zugaktivität des gekäfigten vogels orientieren. Naturwissenschaften 37, 377–378

[B63] Krebs J. R. Biebach H. (1989). Time-place learning by garden warblers (*Sylvia borin*): route or map? Ethology 83, 248–256

[B64] Lewis P. A. Miall R. C. Daan S. Kacelnik A. (2003). Interval timing in mice does not rely upon the circadian pacemaker. Neurosci. Lett. 348, 131–134 10.1016/S0304-3940(03)00521-4 12932811

[B65] Logan J. M. Sanders A. L. Snyder A. Z. Morris J. C. Buckner R. L. (2002). Under-recruitment and nonselective recruitment: dissociable neural mechanisms associated with aging. Neuron 33, 827–840 10.1016/S0896-6273(02)00612-8 11879658

[B66] Lukoyanov N. V. Pereira P. A. Mesquita R. M. Andrade J. P. (2002). Restricted feeding facilitates time-place learning in adult rats. Behav. Brain Res. 134, 283–290 10.1016/S0166-4328(02)00036-0 12191815

[B67] Lyons L. C. Collado M. S. Khabour O. Green C. L. Eskin A. (2006). The circadian clock modulates core steps in long-term memory formation in Aplysia. J. Neurosci. 26, 8662–8671 10.1523/JNEUROSCI.2307-06.2006 16928854 PMC6674367

[B68] Macdonald C. J. Lepage K. Q. Eden U. T. Eichenbaum H. (2011). Hippocampal “time cells” bridge the gap in memory for discontiguous events. Neuron 71, 737–749 10.1016/j.neuron.2011.07.012 21867888 PMC3163062

[B69] McDonald R. J. Hong N. S. Ray C. Ralph M. R. (2002). No time of day modulation or time stamp on multiple memory tasks in rats. Learn. Motiv. 33, 230–252

[B70] Means L. W. Ginn S. R. Arolfo M. P. Pence J. D. (2000). Breakfast in the nook and dinner in the dining room: time-of-day discrimination in rats. Behav. Processes 49, 21–33 10.1016/S0376-6357(00)00068-1 10725650

[B71] Merlin C. Gegear R. J. Reppert S. M. (2009). Antennal circadian clocks coordinate sun compass orientation in migratory monarch butterflies. Science 325, 1700–1704 10.1126/science.1176221 19779201 PMC2754321

[B72] Mistlberger R. E. de Groot M. H. Bossert J. M. Marchant E. G. (1996). Discrimination of circadian phase in intact and suprachiasmatic nuclei-ablated rats. Brain Res. 739, 12–18 10.1016/S0006-8993(96)00466-0 8955919

[B73] Moore D. Siegfried D. Wilson R. Rankin M. A. (1989). The influence of time of day on the foraging behavior of the honeybee, *Apis mellifera*. J. Biol. Rhythms 4, 305–325 10.1177/074873048900400301 2519596

[B74] Mulder C. Gerkema M. P. Van Der Zee E. A. (2010). Circadian time-place association and aging. FENS Forum Eur. Neurosci. 7, 17640. 10.1016/j.bbr.2010.11.039 21115064

[B75] Mulder C. Gerkema M. P. Van der Zee E. A. (2011). The clock genes Per1 and Per2 are not required for memory formation of time-place associations, in 9th Dutch Endo-Neuro-Psycho Meeting, 2.20. Available online at: http://enpmeeting.org/2011/programme.php

[B76] Obrietan K. Impey S. Storm D. R. (1998). Light and circadian rhythmicity regulate MAP kinase activation in the suprachiasmatic nuclei. Nat. Neurosci. 1, 693–700 10.1038/3695 10196585

[B77] Oklejewicz M. Van Der Zee E. A. Gerkema M. P. Daan S. (2001). Memory retention in wild-type and TAU mutant syrian hamsters. Behaviour 138, 789–796 11988228

[B78] Papachristos E. B. Jacobs E. H. Elgersma Y. (2011). Interval timing is intact in arrhythmic Cry1/Cry2-deficient mice. J. Biol. Rhythms 26, 305–313 10.1177/0748730411410026 21775289

[B79] Phan T. X. Chan G. C. Sindreu C. B. Eckel-Mahan K. L. Storm D. R. (2011). The diurnal oscillation of MAP (mitogen-activated protein) kinase and adenylyl cyclase activities in the hippocampus depends on the suprachiasmatic nucleus. J. Neurosci. 31, 10640–10647 10.1523/JNEUROSCI.6535-10.2011 21775607 PMC3146036

[B80] Pittendrigh C. Bruce V. Kaus P. (1958). On the significance of transients in daily rhythms. Proc. Natl. Acad. Sci. U.S.A. 44, 965–973 16590298 10.1073/pnas.44.9.965 PMC528675

[B81] Pizzo M. J. Crystal J. D. (2002). Representation of time in time-place learning. Anim. Learn. Behav. 30, 387–393 12593330 10.3758/bf03195963

[B82] Pizzo M. J. Crystal J. D. (2004). Evidence for an alternation strategy in time-place learning. Behav. Processes 67, 533–537 10.1016/j.beproc.2004.06.004 15519002

[B83] Pizzo M. J. Crystal J. D. (2006). The influence of temporal spacing on time-place discrimination. Learn. Behav. 34, 131–143 16933799 10.3758/bf03193189

[B84] Ralph M. R. Ko C. H. Antoniadis E. A. Seco P. Irani F. Presta C. (2002). The significance of circadian phase for performance on a reward-based learning task in hamsters. Behav. Brain Res. 136, 179–184 10.1016/S0166-4328(02)00131-6 12385803

[B85] Reebs S. G. (1996). Time-place learning in golden shiners (Pisces: *Cyprinidae*). Behav. Processes 36, 253–262 10.1016/0376-6357(96)88023-5 24896874

[B86] Renner M. (1959). Über ein weiteres versetzungsexperiment zur analyse des zeitsinnes und der sonnenorientierung der honigbiene. J. Comp. Physiol. A 42, 449–483

[B87] Rijnsdorp A. Daan S. Dijkstra C. (1981). Hunting in the kestrel, *Falco tinnunculus*, and the adaptive significance of daily habits. Oecologia 50, 391–406 10.1007/BF00344982 28309060

[B88] Sakai T. Tamura T. Kitamoto T. Kidokoro Y. (2004). A clock gene, period, plays a key role in long-term memory formation in Drosophila. Proc. Natl. Acad. Sci. U.S.A. 101, 16058–16063 10.1073/pnas.0401472101 15522971 PMC528738

[B89] Sanada K. Okano T. Fukada Y. (2002). Mitogen-activated protein kinase phosphorylates and negatively regulates basic helix-loop-helix-PAS transcription factor BMAL1. J. Biol. Chem. 277, 267–271 10.1074/jbc.M107850200 11687575

[B90] Shapiro M. L. (2011). Memory time. Neuron 71, 571–573 10.1016/j.neuron.2011.08.006 21867875

[B91] Silver R. Bittman E. L. (1984). Reproductive mechanisms: interaction of circadian and interval timing. Ann. N.Y. Acad. Sci. 423, 488–514 10.1111/j.1749-6632.1984.tb23455.x 6588810

[B92] Staddon J. E. Higa J. J. (1999). Time and memory: towards a pacemaker-free theory of interval timing. J. Exp. Anal. Behav. 71, 215–251 10.1901/jeab.1999.71-215 10220931 PMC1284701

[B93] Stephan F. K. (2002). The “other” circadian system: food as a Zeitgeber. J. Biol. Rhythms 17, 284–292 10.1177/074873040201700402 12164245

[B94] Thorpe C. M. Bates M. E. Wilkie D. M. (2003). Rats have trouble associating all three parts of the time-place-event memory code. Behav. Processes 63, 95–110 10.1016/S0376-6357(03)00051-2 12763272

[B95] Thorpe C. M. Wilkie D. M. (2002). Unequal interval time-place learning. Behav. Processes 58, 157–166 10.1016/S0376-6357(02)00030-X 12044692

[B96] Thorpe C. M. Wilkie D. M. (2007). Rats acquire a low-response-cost daily time-place task with differential amounts of food. Learn. Behav. 35, 71–78 17557393 10.3758/bf03196076

[B97] Valentinuzzi V. S. Neto S. P. D. Carneiro B. T. S. Santana K. S. Arajo J. F. Ralph M. R. (2008). Memory for time of training modulates performance on a place conditioning task in marmosets. Neurobiol. Learn. Mem. 89, 604–607 10.1016/j.nlm.2007.08.002 17904878

[B98] van der Veen D. R. Mulder E. G. Oster H. Gerkema M. P. Hut R. A. (2008). SCN-AVP release of mPer1/mPer2 double-mutant mice *in vitro*. J. Circadian Rhythms 6, 5 10.1186/1740-3391-6-5 18355404 PMC2277380

[B99] Van der Zee E. A. Boersma G. J. Hut R. A. (2009). The neurobiology of circadian rhythms. Curr. Opin. Pulm. Med. 15, 534–539 10.1097/MCP.0b013e3283319b29 19710613

[B100] Van der Zee E. A. Havekes R. Barf R. P. Hut R. A. Nijholt I. M. Jacobs E. H. (2008). Circadian time-place learning in mice depends on Cry genes. Curr. Biol. 18, 844–848 10.1016/j.cub.2008.04.077 18514517

[B101] Van der Zee E. A. Jansen K. Gerkema M. P. (1999). Severe loss of vasopressin-immunoreactive cells in the suprachiasmatic nucleus of aging voles coincides with reduced circadian organization of running wheel activity. Brain Res. 816, 572–579 10.1016/S0006-8993(98)01239-6 9878882

[B102] Van der Zee E. A. Streefland C. Strosberg A. D. Schroder H. Luiten P. G. (1991). Colocalization of muscarinic and nicotinic receptors in cholinoceptive neurons of the suprachiasmatic region in young and aged rats. Brain Res. 542, 348–352 10.1016/0006-8993(91)91590-W 2029643

[B103] Wahl O. (1932). Neue Untersuchungen über das Zeitgedächtnis der Bienen. J. Comp. Physiol. A 16, 529–589

[B104] Wang L. M. Dragich J. M. Kudo T. Odom I. H. Welsh D. K. O'Dell T. J. (2009). Expression of the circadian clock gene Period2 in the hippocampus: possible implications for synaptic plasticity and learned behaviour. ASN Neuro 1:e00012 10.1042/AN20090020 19570032 PMC2695588

[B105] Wansley R. A. Holloway F. A. (1975). Multiple retention deficits following one-trial appetitive training. Behav. Biol. 14, 135–149 1137538 10.1016/s0091-6773(75)90135-2

[B106] Wansley R. A. Holloway F. A. (1976). Oscillations in retention performance after passive avoidance training. Learn. Motiv. 7, 296–302

[B107] Wearden J. H. (2004). Decision processes in models of timing. Acta Neurobiol. Exp. (Wars) 64, 303–317 15283474 10.55782/ane-2004-1515

[B108] Wenger D. Biebach H. Krebs J. R. (1991). Free-running circadian rhythm of a learned feeding pattern in starlings. Naturwissenschaften 78, 87–89

[B109] Widman D. Gordon D. Timberlake W. (2000). Response cost and time-place discrimination by rats in maze tasks. Learn. Behav. 28, 298–309 10.1016/j.beproc.2012.04.004 22542459

[B110] Widman D. R. Sermania C. M. Genismore K. E. (2004). Evidence for time-place learning in the Morris water maze without food restriction but with increased response cost. Behav. Processes 67, 183–193 10.1016/j.beproc.2004.04.001 15240056

[B111] Wilkie D. M. (1995). Time-place learning. Curr. Dir. Psychol. Sci. 4, 85–89

[B112] Wilkie D. M. Carr J. A. R. Siegenthaler A. Lenger B. Liu M. Kwok M. (1996). Field observations of time-place behaviour in scavenging birds. Behav. Processes 38, 77–88 10.1016/0376-6357(96)00026-5 24897632

[B113] Yin B. Troger A. B. (2011). Exploring the 4th dimension: hippocampus, time, and memory revisited. Front. Integr. Neurosci. 5:36 10.3389/fnint.2011.00036 21886612 PMC3154297

[B114] Zheng B. Larkin D. W. Albrecht U. Sun Z. S. Sage M. Eichele G. (1999). The mPer2 gene encodes a functional component of the mammalian circadian clock. Nature 400, 169–173 10.1038/22118 10408444

[B115] Zueger M. Urani A. Chourbaji S. Zacher C. Lipp H. P. Albrecht U. (2006). mPer1 and mPer2 mutant mice show regular spatial and contextual learning in standardized tests for hippocampus-dependent learning. J. Neural Transm. 113, 347–356 10.1007/s00702-005-0322-4 15959842

